# A local review of section 17 leave forms in conjunction with the Mental Health Act Code of Practice: recommendations for future practice

**DOI:** 10.1192/bjb.2024.54

**Published:** 2025-02

**Authors:** Shayanthan Pathmanathan, Georgina Edgerley Harris, Giles Townsend

**Affiliations:** 1 St George's University Hospitals NHS Foundation Trust, London, UK; 2 Surrey and Borders Partnership NHS Foundation Trust, Leatherhead, UK

**Keywords:** Psychiatry and law, in-patient treatment, clinical governance, qualitative research, rehabilitation

## Abstract

**Aims and method:**

The aim of this project was to set out recommendations for the section 17 leave form to reflect guidance provided in the Mental Health Act 1983: Code of Practice, following local Care Quality Commission feedback. We reviewed guidance in the Code and publicly available leave forms to identify items to include in the leave form. Then, we determined which publicly available leave forms included each item and reviewed whether the item should be included in the leave form and whether any reformulation was needed.

**Results:**

Using the method described, we identified a list of items that should be included in the leave form. When comparing the leave forms of different trusts, there was considerable variation with respect to which items were included in each form.

**Clinical implications:**

We provide some recommendations for future practice regarding section 17 leave forms to facilitate consistency with the Code and between different trusts.

Under the Mental Health Act 1983,^1^ ‘leave’ is the absence from hospital, for a patient detained under the Act, subject to such conditions (if any) as the responsible clinician considers necessary in the interests of the patient or for the protection of others. Patients who are detained under the Act can only leave, irrespective of the duration of the leave, if they are granted leave of absence under section 17 of the Act.

Leave is frequently used in the management of patients held under the Act and is assumed to have therapeutic benefits and assist with the return of patients back into the community,^2^ despite a lack of evidence in the literature.^3^ The potential benefits include better symptom outcomes, assessment of risk on discharge, testing rehabilitation and recovery^4–6^ and ensuring the least restrictive practice, as well as maximising independence, one of the five guiding principles of the Mental Health Act 1983: Code of Practice.^7^ Furthermore, leave can assist patients in maintaining and enhancing relationships and social networks.^8,9^

However, granting leave also carries risks; a fifth of all in-patient suicides occurred during authorised leave in a case–control study in England,^10^ whereas other studies have shown that leave can lead to worse patient outcomes, such as increased length of stay and psychiatric readmissions.^11,12^ Responsible clinicians should also be mindful that leave can be used inappropriately as part of defensive practice, when discharge or community treatment orders (CTOs) may be more appropriate.^13,14^ Ultimately, the decision to grant leave must be based on a robust risk assessment.

Despite the ubiquitous use of leave, research on the implementation and effectiveness of leave remains surprisingly limited.^3,15^ More literature is available on unauthorised leave, perhaps unsurprisingly, given the increased risk to the patient and others and the reputational damage to the institution in such situations.^16^ For authorised leave, research has focused on forensic as opposed to civil settings,^3^ possibly reflecting the more explicit guidance for leave in a forensic setting. For civil leave, there is currently no national guidance for a standardised approach to leave policies; instead, guidance is usually provided by local trust policies based on the Code. Barlow et al^3^ found that decisions on leave were often based on heuristic rules, rather than being evidence based.

Guidance on implementation of the Act is provided by the Mental Health Act 1983: Code of Practice^7^ in England and Mental Health 1983 Code of Practice for Wales Review^17^ in Wales. Chapter 27: Leave of Absence of the Code sets out the best practice for implementing section 17 leave to ensure consistency with the Act. The guidance provided is very much on the implementation of the leave, and the only direct guidance on recording leave is that there should be a standardised system for the responsible clinician to record the leave and specify conditions attached to the leave. This is nearly always achieved through completion of the section 17 leave form, which is usually part of a trust's section 17 leave policy. An audit^15^ found that leave policies across the sector were highly inconsistent, which is likely to lead to differences in implementation of leave and the associated outcomes, creating inequities between patients.

Following a Care Quality Commission inspection at the Meadows Old Age Psychiatry Inpatient Unit, within Surrey and Borders Partnership NHS Foundation Trust, the medical team reviewed and updated the trust's leave form,^18^ which is part of the trust's leave policy.^19^ The key issues raised about the leave form were around the generic nature of the conditions of the leave, the lack of precision around the frequency of leave and the omission of explicit review dates in some cases.

The aim of this quality improvement project was to suggest recommendations for the local leave form, primarily to reflect the guidance provided in the Code of Practice, with the intention that these recommendations could then be applicable more generally to non-restricted in-patient units in England and Wales.

## Method

Potential guidelines for restricted patients or for mental health trusts outside England and Wales were not considered as part of this work. We also did not consider guidelines for leave polices more broadly, focusing purely on guidelines for leave forms.

We aimed to create a list of items that should be included in the leave form. The starting point for these items was the ‘Leave of absence’ chapter in the Code, which provided guidance on the implementation of section 17 leave. We read each subsection of the ‘Leave of absence’ chapter of the Code in turn and identified items to include in the leave form that would document either explicit or implicit compliance with the Code.

In addition to the Code, we reviewed the publicly available leave forms of other mental health trusts to identify items to include that could facilitate good practice despite not necessarily being required to evidence compliance with the Code. We reviewed the leave policies and forms of mental health trusts in the south-east and London, which were geographically closest to Surrey and Borders Partnership NHS Foundation Trust. Those trusts for which the information was available publicly were included in this review; these comprised Hertfordshire Partnership University,^20^ Solent,^21^ Sussex Partnership,^22^ Camden and Islington,^23^ and Central and North West London.^24^

Given the limited literature on implementation of leave,^3,15^ we also used the clinical experience of the responsible clinician on the ward to identify any other items that might be helpful to include. However, this did not result in identification of any further items for inclusion, as we had already interrogated the ‘Leave of absence’ chapter of the Code and numerous trusts’ leave forms, including our own local leave form.

Using the full list of items identified, we first evaluated which of the trusts’ leave forms included each item. We then reviewed each of the items and considered whether these were included in most of the forms, and, if not, whether it would be appropriate to require that item to be addressed within the leave form. In addition, we also considered whether any reformulation of the requirements was needed to reflect current practice.

Based on this finalised list of requirements for the leave form, we then formulated recommendations to facilitate the implementation of the requirements identified and to promote consistency in the approach taken by different trusts.

## Results

Table 1 below sets out the items identified for inclusion in the leave form and the origin of each item.

**Table 1:** Items for inclusion in leave form

Item	Source
Declaration that risk assessment has been completed and conditions of leave can be met	CoP
Explanation of why a CTO is not used for leave of more than 7 days	CoP
Separate sections for details of short-term leave and longer periods of leave	CoP
Inclusion of the review date for the leave	CoP
Standard statement that leave is conditional on patient's health not deteriorating significantly	CoP
Section for location and other conditions of leave	CoP
Escorted/accompanied/unaccompanied given as tick-box options	CoP
Check whether detention period finishes during the leave granted	CoP
Inclusion of emergency contact details	Leave forms
Immediate actions to take if patient does not return from leave or absconds from escort or accompanying person	Leave forms
Option for leave for urgent treatment in leave form	Leave forms

CoP, Code of Practice;^7,17^ Leave forms, analysis of leave forms.^18,20–24^

### Analysis of the code

Details of the analysis of the Code are presented below. Subsections of the chapter that are not applicable to the leave form (‘Care and treatment while on leave’, ‘Patients who are in hospital but not detained’) or are only applicable to restricted patients (‘Restricted patients’) were not included in the analysis.

#### General points

Formal authorisation for leave is only required for leave outside the ‘grounds of the hospital’; hence, there should be a clear definition of what constitutes the grounds of the hospital. However, this definition should be set out in the leave policy rather than in the leave form^15^ and so does not impose any requirements on the leave form.

#### Powers to grant leave

When granting leave, the responsible clinician should consider the risks and benefits of approving or refusing leave, as well as any conditions to be attached to the leave, and undertake a risk assessment and put in place any necessary safeguards. The Code also states that the responsible clinician should establish that any conditions, such as accommodation, can be met. Although a declaration of risk assessment completion will not necessarily provide any assurance on the quality of the risk assessment, having an explicit statement in the leave form should, in the authors’ view, encourage a better quality of risk assessment. For periods of leave consisting of more than 7 consecutive days, the Code recommends that the responsible clinician consider whether a CTO is more appropriate.

#### Short-term leave and longer periods of leave

The Code has separate sections for ‘short-term leave’ and ‘longer periods of leave’, although no precise definitions of these terms is provided. For both leave types, there should be regular reviews of the authorised leave. The Code states that the responsible clinician can authorise others to manage the implementation of short-term leave. This flexibility should be documented in the leave form, although it seems more sensible to fit this into the ‘Conditions of leave’ section of the leave form, as discussed below under ‘Recording of leave’.

The Code also specifies that for both short-term leave and longer periods of leave, the responsible clinician should state any circumstances when the leave should not go ahead, for example, if the patient's health has deteriorated significantly since the time the leave was authorised.

#### Recording of leave

The leave form satisfies the Code's recommendation for a standardised system for the responsible clinician to record the leave granted under section 17. The recording system should specify the conditions of the leave approved by the responsible clinician. This can cover numerous areas such as the places the patient can go to and details of the implementation of leave by the wider team.

#### Escorted leave and accompanied leave

There are separate subsections in the Code for ‘escorted leave’ and ‘accompanied leave’, which we discuss together. Both escorted and accompanied leave can be imposed as conditions of the leave, with a description of both leave types provided in the Code. Given this guidance, it seems appropriate to have options in the leave form to choose either escorted, accompanied or unaccompanied leave.

#### Leave to reside in other hospitals

It may be necessary to transfer patients to other healthcare facilities to provide treatment. This leave would in theory require formal section 17 approval; however, in emergency situations, the leave form often cannot be completed in time. The procedures to follow in these circumstances should be set out in the leave policy.

#### Renewal of authority to detain

The Code states that it is possible to renew a patient's detention under the Act while the patient is on leave, and that leave should not be used as an alternative to discharging the patient. Therefore, it would be sensible to have a check in the leave form regarding whether the patient's detention finishes during the leave period.

### Analysis of leave forms

The contents of the leave forms included in the analysis were to some extent based on guidance from the Code, limiting the additional items identified from this part of the analysis. One feature in two of the forms was the inclusion of emergency contact details in case any issues arise. Inclusion of emergency contact details on the leave form appears sensible and practical, especially as a copy of the form is given to the patient and any carers. Two of the trusts also include a section in the leave form for an action plan in case the patient does not return from leave or absconds from the escort or accompanying person, which provides guidance for those involved in the care of the patient when on leave.

With respect to leave for urgent treatment, leave polices usually require section 17 leave approval and completion of the leave form as soon as possible, which may be after the leave has commenced. In one of the leave forms, there was an option for indicating that the leave was for urgent treatment.

### Comparison of leave forms with requirements

Table 2 provides a summary of the comparison of leave forms of different trusts, with respect to the required features identified in Table 1. As shown in the table, there was considerable variation regarding which of the items were included and how such items were addressed within the leave form.

**Table 2:** Comparison of leave forms across mental health trusts

	Surrey and Borders Partnership	Hertfordshire Partnership University	Solent	Sussex Partnership	Camden and Islington	Central and North West London
Declaration that risk assessment has been completed and conditions of leave can be met	Yes	No	No	No	Partially	Partially
Explanation of why a CTO is not used for leave of more than 7 days	Partially	Partially	Yes	Yes	Yes	Yes
Separate sections for details of short-term leave and longer periods of leave	Partially	No	Yes	Partially	No	Yes
Inclusion of the review date for the leave	No	No	Yes	Yes	No	Yes
Standard statement that leave is conditional on patient's health not deteriorating significantly	Partially	No	Partially	No	No	No
Section for location and other conditions of leave	Yes	Yes	Partially	Partially	Partially	Yes
Escorted/accompanied/unaccompanied given as tick-box options	Yes	Yes	Partially	Yes	No	Yes
Check whether detention period finishes during leave	No	No	No	No	No	No
Inclusion of emergency contact details	No	No	Yes	Yes	No	No
Immediate actions to take if patient does not return from leave or absconds from escort or accompanying person	No	No	No	No	Yes	Yes
Option for leave for urgent treatment in leave form	No	No	Yes	No	No	No

## Discussion

The leave form provides patient- and situation-specific documentation of each leave and is used universally across the sector to evidence compliance with the Code. Documentation is a key element of good governance and internal controls; thus, leave forms are a critical element of granting leave of absence. Completion of the leave form is often the final stage of leave authorisation and acts as a safety net for the whole leave process. Therefore, it is somewhat surprising that there is so much variation in the content of leave forms across mental health trusts.

Only a minority of leave forms have statements that the various requirements set out in the Code have been considered, and only one form had a statement that in the opinion of the responsible clinician, the conditions of the leave could indeed be met. In our view, it is critical to explicitly state that that the risks and benefits of granting leave have been considered, and that the conditions of the leave can be implemented. Similarly, the statement that leave is conditional on the patient's health not deteriorating significantly was frequently missing from the leave forms; we suggest that this should be an explicit part of the ‘Conditions of leave’ section.

There was variability in how the conditions of leave were set out, with many forms not having a separate section for the locations that the patient could visit. We recommend that there is a separate section to set out the location or areas that can be visited, in addition to a section for the general conditions of leave.

There were also notable differences in documentation of the different leave types. Some trusts did not separate short-term and longer periods of leave, whereas one trust classified short-term leave as anything less than 7 days rather than leave not requiring an overnight stay. Most trusts did explicitly state an end date for short-term leave, indicating that it is good practice to include an end date for recurring short-term leave. Reflecting this, it would be appropriate to have separate sections for leave without overnight stay and leave with overnight stay, rather than using short-term and longer periods of leave.

Finally, although none of the forms defined whether the detention period finished during the leave, we recommend that this is an explicit item in the leave form, to ensure this aspect is not overlooked when granting leave.

Here, we set out the reformulated recommendations for the leave form to implement the requirements identified in this article and to encourage consistency between leave forms across different mental health trusts.

- Include statements in the leave form confirming that risk assessment has been performed and that the responsible clinician considers that the conditions of leave can be met.
- Provide separate sections for leave without overnight stay (short-term leave) and leave with overnight stay (longer period of leave) in the leave form, with details of:
  - leave without overnight stay: duration of leave, frequency of leave, start and end dates for period over which short-term leave is given;
  - leave with overnight stay: start and end times and dates of the leave and if the period is longer than 7 consecutive days, then explanation as to why CTO has not been used.
- Include review date for all types of leave.
- Provide section in leave form to set out the location or area of leave, including some of the standard local areas that can be visited.
- Provide section in leave form to set out other conditions of leave and consider having a standard statement that one condition of leave is that the patient's health has not deteriorated significantly since authorisation.
- Provide options for escorted, accompanied or unaccompanied leave for leave without overnight stay.
- Provide section for escorting and accompanying requirements giving more information on these requirements. This section can also include escorting and accompanying requirements that may be requested for leave with overnight stay, such as visits to other hospitals.
- Include confirmation of whether expiry of detention period occurs during the leave period.
- Provide emergency contact details at the start of the leave form in case of any problems during leave.
- Include immediate action plan if patient absconds or does not return at the end of leave.
- Provide section in leave form to state whether form is being completed retrospectively owing to leave for urgent treatment.

These recommendations for the leave form could enhance the controls in place and help facilitate better and more consistent practice, leading to more equitable outcomes among patient groups. In the authors’ opinion, a bottom-up approach of changing leave forms across trusts is likely to be much easier and quicker to implement than a top-down approach of updating leave policies. However, we acknowledge that the leave form is only one element of several interrelated processes in granting leave that include the risk assessment and risk management strategies such as a detailed absence without leave plan.

## About the authors

**Shayanthan Pathmanathan** is an FY1 at St George's University Hospitals NHS Foundation Trust, London, UK. **Georgina Edgerley Harris** is an ST5 at Surrey and Borders Partnership NHS Foundation Trust, Leatherhead, UK. **Giles Townsend** is a consultant psychiatrist at Surrey and Borders Partnership NHS Foundation Trust, Leatherhead, UK.
